# Efficacy of a Web App for Cognitive Training (MeMo) Regarding Cognitive and Behavioral Performance in People With Neurocognitive Disorders: Randomized Controlled Trial

**DOI:** 10.2196/17167

**Published:** 2020-03-11

**Authors:** Philippe Robert, Valeria Manera, Alexandre Derreumaux, Marion Ferrandez Y Montesino, Elsa Leone, Roxane Fabre, Jeremy Bourgeois

**Affiliations:** 1 Cognition Behaviour Technology Lab Université Côte d'Azur Nice France; 2 Association Innovation Alzheimer Nice France; 3 Centre Memoire Centre Hospitalier Universitaire de Nice Nice France; 4 Departement de Santé Publique Centre Hospitalier Universitaire de Nice Nice France

**Keywords:** neurocognitive disorders, Alzheimer disease, cognition, motivation, apathy, intervention

## Abstract

**Background:**

Cognitive and behavioral symptoms are the clinical hallmarks of neurocognitive disorders. Cognitive training may be offered to reduce the risks of cognitive decline and dementia and to reduce behavioral symptoms, such as apathy. Information and communication technology approaches, including serious games, can be useful in improving the playful aspect of computerized cognitive training and providing motivating solutions in elderly patients.

**Objective:**

The objective of this study was to assess the effectiveness of employing the MeMo (Memory Motivation) Web app with regard to cognitive and behavioral symptoms in patients with neurocognitive disorders.

**Methods:**

MeMo is a Web app that can be used on any Web browser (computer or tablet). The training activities proposed in MeMo are divided into the following two parts: memory and mental flexibility/attention. The study included 46 individuals (mean age 79.4 years) with a diagnosis of neurocognitive disorders at the Institut Claude Pompidou Memory Center in Nice. This randomized controlled study compared the evolution of cognition and behavior between patients not using MeMo (control group) and patients using MeMo (MeMo group) for 12 weeks (four sessions per week). Patients underwent memory and attention tests, as well as an apathy assessment at baseline, week 12 (end of the training period), and week 24 (12 weeks after the end of the training sessions). In addition, to assess the impact of high and low game uses, the MeMo group was divided into patients who used MeMo according to the instructions (about once every 2 days; active MeMo group) and those who used it less (nonactive MeMo group).

**Results:**

When comparing cognitive and behavioral scores among baseline, week 12, and week 24, mixed model analysis for each cognitive and behavioral score indicated no significant interaction between testing time and group. On comparing the active MeMo group (n=9) and nonactive MeMo group (n=13), there were significant differences in two attention tests (Trial Making Test A [*P*=.045] and correct Digit Symbol Substitution Test items [*P*=.045]) and in the Apathy Inventory (AI) (*P*=.02). Mixed analysis (time: baseline, week 12, and week 24 × number of active days) indicated only one significant interaction for the AI score (*P*=.01), with a significant increase in apathy in the nonactive MeMo group.

**Conclusions:**

This study indicates that the cognitive and behavioral efficacies of MeMo, a Web-based training app, can be observed only with regular use of the app. Improvements were observed in attention and motivation.

**Trial Registration:**

ClinicalTrials.gov NCT04142801; https://clinicaltrials.gov/ct2/show/NCT04142801

## Introduction

Memory, attention, and behavioral symptoms are the clinical hallmarks of neurocognitive disorders (NCDs), such as Alzheimer disease and related disorders [1]. While there is no curative treatment for major NCDs, the proactive management of modifiable risk factors can delay or slow down the onset or progression of these diseases [2]. In particular, a number of randomized controlled trials (RCTs) suggested that lifestyle-based multidomain interventions, including management of cardiovascular risk factors and cognitive training, may be feasible and effective in reducing the risk of cognitive decline in older at-risk adults [3]. Cognitive training, which refers to the guided practice of specific standardized tasks designed to enhance particular cognitive functions [4], appears to be a promising option to improve cognitive performance in older adults [5]. For this reason, the World Health Organization guidelines for risk reduction of cognitive decline and dementia indicate that cognitive training may be offered to older adults with normal cognition and with mild cognitive impairment to reduce the risks of cognitive decline and dementia [6]. However, evidence on the efficacy of cognitive training in people with cognitive decline (minor and major NCDs) is still scarce, and there is a lack of RCTs [5,7,8]. Furthermore, most existing studies only focused on the improvement of cognitive function. In NCDs, cognitive symptoms are often associated and are sometimes even preceded by behavioral symptoms, such as apathy [9]. Apathy is a multidimensional syndrome characterized by a significant reduction in goal-directed activity [10,11]. It represents the most common behavioral and psychological symptoms in people with Alzheimer disease and is often observed in Parkinson disease and other dementia-related disorders, such as vascular dementia and frontotemporal dementia [12]. Pharmacological therapies have demonstrated limited efficacy in the management of apathy associated with NCDs and neuropsychiatric conditions [13]. However, nonpharmacological treatments, including cognitive training via serious games and virtual reality, are considered promising approaches for apathy management in people with NCDs [14-16].

The use of information and communication technology (ICT) in the health domain is progressively expanding. Recently, increasing attention is being devoted to the field of NCDs, where ICT is employed to both support and improve the assessment of different functional and cognitive abilities [17-20] and to provide alternative solutions for patient stimulation and rehabilitation. Serious games are mental and physical contests played with a computer in accordance with specific rules, which use entertainment to promote training, education, health, public policy, and strategic communication objectives [21]. One of the targets is to improve the playful aspect of computerized cognitive training in order to improve motivation [22]. ICT can also be useful in providing solutions that can be proposed to patients with NCDs coming for consultations to memory centers. Cognitive training is indeed indicated for some of them according to clinician judgement, but it is often difficult to propose. This is due to several reasons, including logistic constraints (eg, necessity to visit the memory center several times a week to participate in stimulation sessions and clinician availability), the necessity to pay subscription fees, the fact that the effectiveness of existing tools is not scientifically proven, and the fact that most existing free training platforms are available only in English. To overcome these problems, the Alzheimer Innovation Association and the CoBTeK research team located in the Nice Memory Center at the Institut Claude Pompidou have developed MeMo (Memory Motivation), a free multilingual Web app that can be used at home by patients. This Web app has been designed by a multidisciplinary team of neuropsychologists, physicians, engineers, and Web designers. The objective of this study was to assess the effectiveness of employing the MeMo platform with regard to cognitive performance and apathy in patients with mild and major NCDs.

## Methods

### Web App

MeMo is a Web app that can be used on any Web browser (computer or tablet). On a tablet, the app can be installed on the home screen to look like a native app and can be used offline. Users first create an account, so their progress can be logged in the database and visualized in the form of charts. Clinicians can create professional accounts and follow the game progression of all patients who agree to be added to their professional lists.

The app was developed in HTML5/CSS3 with PHP5/MySQL on backend.gnosis. The platform opened in April 2015. In November 2019, 9654 accounts had been created and 327,838 exercises had been performed. MeMo is accessible at the following URL: https://www.memory-motivation.org/home-2/. It is available in French and English, and an Italian version is in development.

The exercises were designed by neuropsychologists and medical doctors. Every exercise was conceived to train a specific cognitive function in order to allow personalized training according to observed deficits. The training activities proposed in MeMo are divided into two parts. The first part involves memory, which includes the following three activities: “recognition” for visual memory training, “MeMo quiz” for working memory training, and “faces” for associative memory training. The second part involves mental flexibility/attention, which includes the following three activities: “arrows” for processing speed, inhibitory control, and mental flexibility training; “tricky cards” for working memory training; and “jumping squares” for reaction anticipation and inhibitory control training. The exercises were conceived to try to motivate people to play. Based on recommendations published by our research group [22], the exercises were designed to be adapted to the target population (eg, simple rules, clear and simplified graphical user interface, clear feedback on performance, instruction reminders, etc), and they embed game rules to include challenges for the player and allow progression in exercise difficulty according to performance.

After creating an account, users can track the evolution of their performance in the exercises. This also allows therapists to follow the evolution of patient performance over time. Indeed, each exercise has several levels of difficulty. Whenever users obtain the maximum score at one level, they are advanced automatically to the next level.

### Study Design and Population

This study aimed to compare the evolution of cognitive performance, as well as the motivational status (presence of apathy) between participants using the Web app (MeMo group) and participants not using it (control group). Participants were recruited at the Nice Memory Center located in the Institut Claude Pompidou. The memory center receives approximatively 1700 patients per year. Consultations are performed by a multidisciplinary team of medical doctors (two psychiatrists, one neurologist, and one geriatrician), five neuropsychologists, and one speech therapist. Inclusion in the MeMo study was proposed by all the staff members. All participants provided informed written consent before starting the study. The study was performed in compliance with the Declaration of Helsinki and was approved by the ethics committee of CCP Sud Ed IV-Montpellier (2016-A00879-42). Individuals were included if they were outpatients consulting with the memory center, were older than 60 years, had a Mini-Mental State Examination (MMSE) [23] score between 16 and 28/30, and met the Diagnostic and Statistical Manual of Mental Disorders, Fifth Edition diagnostic criteria for mild or major NCDs. Patients were not included if they were not able to read and write in French, had a hearing or major visual impairment, had a history of premorbid intellectual disability, had already used the MeMo app, and were presently involved in structured memory training activities.

The study included 49 participants. Three of them were included incorrectly, and they have not been included in the population description. Thus, the final sample included 46 participants (20 with mild NCDs and 26 with major NCDs) [24]. In terms of disorder etiology, 32 participants were diagnosed with probable Alzheimer disease and 14 with mixed disorders. Among the 46 participants, 42 had a concomitant pharmacological treatment (memantine or cholinesterase inhibitors). Additionally, 25 had concurrent nonpharmacological interventions, including speech therapy (n=22) and other noncognitive interventions, such as kinesitherapy and musicotherapy (n=8).

Of the 46 patients, 25 were randomized to the MeMo group (11 with mild NCDs and 14 with major NCDs) and 21 were randomized to the control group (9 with mild NCDs and 12 with major NCDs). Patients underwent a neuropsychological and behavioral assessment battery at baseline, week 12 (end of the training period), and week 24 (12 weeks after the end of the training sessions). The assessments included the MMSE and Informant Questionnaire on Cognitive Decline in the Elderly (IQCODE). The IQCODE is widely used as a complementary screening tool. The 26 items of the instrument were proposed in the following two versions: informant version (IQCODEi) and self-completion version (IQCODEs) [25]. Each item is rated on a 5-point scale from 1 (much better) to 5 (much worse), and the ratings are averaged over the 26 items to give a score from 1 to 5, with 3 representing no change in any item. Neuropsychological assessments included the Free and Cued Selective Reminding Test [26] for episodic memory and a set of attention tasks [27], including the Trial Making Test A (TMT A), Stroop test, Digit Symbol Substitution Test (DSST), and Frontal Assessment Battery (FAB) [28]. Behavioral symptoms were assessed using the Neuropsychiatric Inventory (NPI) [29], including the apathy subscale, and the Apathy Inventory (AI; clinician version) for quantitative apathy assessment [30].

Participants randomized to the MeMo group were instructed on how to use the app in the clinic by a researcher involved in the study and were asked to train for 12 weeks. Participants were asked to train regularly, with four sessions of 30 minutes each per week. In every session, participants were asked to perform the following six exercises for 5 minutes each (presented in randomized order): “recognition” for visual memory training; “MeMo quiz” for working memory training; “faces” for associative memory training; “arrows” for processing speed, inhibitory control, and mental flexibility training; “tricky cards” for working memory training; and “jumping squares” for reaction anticipation and inhibitory control training. All exercises started at level 1, and the difficulty was progressively adjusted, so that when participants completed one level, they progressed to the next (during the same session or the following session if time expired). The order of presentation of the exercises was randomized for each session and automatically proposed by the app. During the training, participants could contact the study investigators at any time in case of technical problems or questions about the use of the app. All participants used the French version of the app. During the trial, the medical doctor responsible for the participants had no access to the game results (participants were assigned with anonymous account codes). Neuropsychological assessments at baseline, week 12, and week 24 were performed by a neuropsychologist blinded to the participant group allocation (MeMo and control groups).

### Statistical Analysis

Data are presented as mean (SD) for quantitative variables and as frequency and percentage for qualitative variables (sex, education level, diagnosis, and concomitant treatment). Comparisons between the different groups were performed using the Student *t*-test or Wilcoxon-Mann-Whitney test for quantitative variables and the χ^2^ or Fisher exact test for qualitative variables. Paired Wilcoxon tests were used to assess the evolution of scores between different times 2 to 2 (baseline, week 12, and week 24) for each diagnosis of cognitive impairment. Mixed models taking into account data from the same patient were used to assess the interaction (time × group). These results were adjusted for age and sex. All scores were used as the dependent variable. In the active MeMo group, an additional analysis was performed according to the instruction that the app had to be used regularly. A *P* value <.05 was considered significant, and 95% CIs were indicated. The analyses were performed using the free software R 3.5.1 (The R Foundation for Statistical Computing, Vienna, Austria).

## Results

As presented in Table 1, no significant differences in demographics, diagnosis, and clinical characteristics were found between the control group and MeMo group.

On comparing cognitive and behavioral scores among baseline, week 12, and week 24, there were no significant differences in the control group. In the MeMo group, the TMT A time score significantly increased (66.2 s at baseline to 78.7 s at week 12; *P*=.02), suggesting a slight decrease in participants’ attention abilities over the 12 weeks, which is compatible with the fact that participants had neurodegenerative disorders. A slight increase in the total NPI score was also found (8.9 s at baseline to 11.3 s at week 12), but this was almost negligible considering that the NPI score can range from 0 to 144 s. Mixed model analysis for each cognitive and behavioral score indicated no significant interaction between the testing time and group.

In order to better explore the MeMo group, we checked whether participants adhered to the proposed study protocol. Data for 22 participants were retrieved directly from the app. For one participant, the data could not be recovered at week 12 (no update on the tablet), and another participant had an adverse event at week 12. The number of days of use varied from 1 day to 78 days over a maximum period of 80 days. The same variability was observed in the number of games played (mean 527 [SD 661]). Therefore, to better assess the impact of high and low game uses, the population was divided into participants who used MeMo according to the instructions (about once every 2 days; active MeMo group) and those who used it less (nonactive MeMo group). A 40-day threshold was selected because it is close to the number of active days described by the protocol, and moreover, it appropriately splits the participants into two groups (<40 active days for the nonactive MeMo group and 40-78 active days for the active MeMo group). The two groups were not different in terms of age, sex, level of education, presence of concomitant treatments, or results at baseline (NPI, *P*=.45; AI, *P*=.69; and IQCODE, *P*=.39).

Table 2 shows the cognitive and behavioral score changes between baseline and week 12. Significant differences between the active and nonactive MeMo groups were found in two attention tests (TMT A and correct DSST items) and in the AI. Specifically, participants in the nonactive MeMo group showed decreased performance in attention (longer reaction times in the TMT A) and became slightly more apathetic (higher scores in AI) over the 12 weeks as compared with participants in the active MeMo group, who showed stable results over the training duration. Furthermore, participants in the active MeMo group showed improved performance in the DSST over the training, whereas participants in the nonactive MeMo group showed stable results over time.

**Figure 1:**
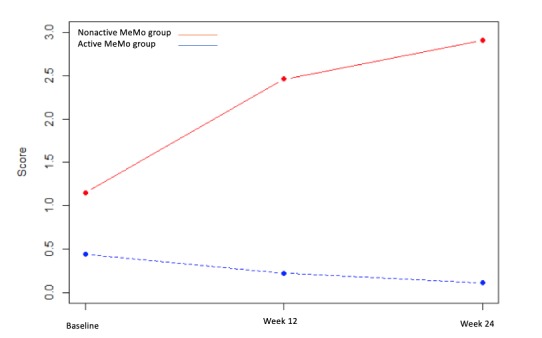
Apathy Inventory score changes in the active MeMo group and nonactive MeMo group. MeMo: Memory Motivation.

Mixed analysis (time: baseline, week 12, and week 24 × number of active days) indicated only one significant interaction for the AI score (*P*=.01), with a significant increase in apathy in the nonactive MeMo group at week 12 (adjusted coefficient=1.31, 95% CI 0.56-2.05, *P*=.001) and at week 24 (adjusted coefficient=1.61, 95% CI 0.82-2.41, *P*<.001). The results are illustrated in Figure 1.

Table 3 shows the highest performance levels observed for each MeMo game at week 12. For each game, participants in the active MeMo group achieved a higher level of performance as compared with participants in the nonactive MeMo group (*P*<.02).

Finally, correlations between the evolution of cognitive performance (differences in the scores between baseline and week 12) and the MeMo activities were tested. The only significant correlations were between the score changes in the FAB and the number of activities played (Spearman ρ=0.43, *P*=.047) and number of active minutes (Spearman ρ=0.48, *P*=.02). Additionally, more game play by the MeMo participants (number of active days and number of active minutes) was associated with a higher difference in the FAB scores between posttraining and pretraining performance.

**Table 1:** Demographics and clinical characteristics of the study participants.

Characteristic		Overall population (n=46)	Control group (n=21)	MeMo^a^ group (n=25)	*P* value^b^
Age, mean (SD)		79.4 (6.8)	78.8 (6.6)	79.8 (7.0)	.63
**DSM-5^c^ diagnosis, n (%)**					.94
	Major NCDs^d^	26 (56)	12 (57)	14 (56)	
	Minor NCDs	20 (43)	9 (42)	11 (44)	
**Sex, n (%)**					.07
	Female	24 (52)	14 (66)	10 (40)	
	Male	22 (47)	7 (33)	15 (60)	
**Level of education, n (%)**					.82
	Primary level	10 (21)	4 (19)	6 (24)	
	Secondary level	21 (47)	12 (57)	10 (40)	
	Superior level	14 (30)	5 (23)	9 (36)	
MMSE^e^, mean (SD)		21.4 (3.5)	21.6 (2.7)	21.3 (4.1)	.70
IQCODE^f^ patient, mean (SD)		83.8 (5.7)	82.5 (4.8)	84.9 (6.2)	.22
IQCODE caregiver, mean (SD)		93.0 (8.7)	91.3 (8.9)	94.1 (8.6)	.44
FAB^g^, mean (SD)		13.0 (2.2)	13.4 (2.0)	12.6 (2.2)	.18
FCSRT^h^ immediate recall, mean (SD)		13.4 (4.1)	13.6 (4.6)	13.2 (3.7)	.81
FCSRT delayed recall, mean (SD)		1.3 (2.1)	1.0 (1.1)	1.6 (2.6)	.73
TMT A^i^, mean (SD)		65.7 (25.7)	64.7 (24.7)	66.6 (27.0)	.95
STROOP^j^, mean (SD)		3.0 (1.0)	2.7 (0.9)	3.3 (1.1)	.049
DSST^k^, mean (SD)		32.3 (12.6)	36.3 (13.2)	29.0 (11.3)	.08
NPI^l^, mean (SD)		8.2 (8.3)	8.3 (9.4)	8.3 (7.6)	.72
AI^m^, mean (SD)		0.7 (1.6)	0.7 (1.3)	0.8 (1.8)	.97

^a^MeMo: Memory Motivation.

^b^Comparisons performed using the Student *t*-test for quantitative variables and the χ^2^ or Fisher exact test for qualitative variables.

^c^DSM-5: Diagnostic and Statistical Manual of Mental Disorders, Fifth Edition.

^d^NCDs: neurocognitive disorders.

^e^MMSE: Mini-Mental State Examination.

^f^IQCODE: Informant Questionnaire on Cognitive Decline in the Elderly.

^g^FAB: Frontal Assessment Battery.

^h^FCSRT: Free and Cued Selective Reminding Test.

^i^TMT A: Trail Making Test A; score, time in seconds.

^j^STROOP: Stroop test.

^k^DSST: Digit Symbol Substitution Test.

^l^NPI: Neuropsychiatric Inventory.

^m^AI: Apathy Inventory.

**Table 2:** Cognitive and behavioral score changes between baseline and week 12 in the active MeMo group and nonactive MeMo group.

Test/scale	Score difference^a^		*P* value^b^
	Nonactive MeMo^c^ group (active days <40; n=13)	Active MeMo group (active days ≥40; n=9)	
MMSE^d^	−0.8 (1.8)	−0.7 (2.0)	.84
IQCODE^e^ patient	−1.9 (2.8)	−1.2 (4.4)	.46
IQCODE caregiver	0.9 (5.9)	3.8 (2.2)	.39
FAB^f^	−0.2 (2.4)	0.7 (2.4)	.33
FCSRT^g^ immediate recall	1.4 (3.0)	1.24.6)	.79
FCSRT delay recall	−0.6 (1.3)	−0.2 (2.1)	.28
TMT A^h^	21.5 (29.9)	0.3 (14.8)	.045
STROOP^i^	−0.1 (1.0)	0.2 (1.5)	.84
DSST^j^	0.5 (7.9)	4.25.1)	.04
NPI^k^	6.8 (9.7)	2.3 (5.1)	.45
AI^l^	1.3 (1.5)	−0.2 (1.1)	.02

^a^Values are presented as mean (SD).

^b^Student *t*-test.

^c^MeMo: Memory Motivation.

^d^MMSE: Mini-Mental State Examination.

^e^IQCODE: Informant Questionnaire on Cognitive Decline in the Elderly.

^f^FAB: Frontal Assessment Battery.

^g^FCSRT: Free and Cued Selective Reminding Test.

^h^TMT A: Trail Making Test A; score, time in seconds.

^i^STROOP: Stroop test.

^j^DSST: Digit Symbol Substitution Test.

^k^NPI: Neuropsychiatric Inventory.

^l^AI: Apathy Inventory.

**Table 3:** Maximum performance score observed for each game in the MeMo app at the end of the study after 12 weeks of use.

Game (maximum possible score)	Maximum performance score^a^		*P* value^b^
	Nonactive MeMo group (active days <40; n=13)	Active MeMo group (active days ≥40; n=9)	
Recognition (10)	3.3 (1.8)	6.2 (2.3)	.007
Quiz (8)	3.3 (2.1)	5.7 (1.7)	.02
Faces (10)	4.2 (2.9)	7.6 (1.1)	.003
Arrows (10)	4.4 (2.6)	8.9 (1.2)	.001
Tricky cards (9)	3.8 (0.7)	5.0 (1.2)	.007
Jumping square (12)	5.1 (2.4)	9.8 (1.5)	<.001

^a^Values are presented as mean (SD).

^b^Wilcoxon-Mann-Whitney test.

## Discussion

Cognitive training is regarded as a promising option to slow cognitive decline in the elderly [5] and to improve cognitive function and behavioral symptoms in people with neurodegenerative disorders [7,8]. However, RCTs focused on the efficacy of cognitive training in people with mild and major NCDs having cognitive and behavioral symptoms are still lacking [5]. Furthermore, most existing studies only investigated improvements in cognitive function. Behavioral symptoms, such as apathy (disorder of goal-directed behavior), depression, and agitation, are very common in people with NCDs, and they greatly contribute to increased caregiver burden and reduced patient quality of life [31]. Given that pharmacological treatments have shown limited efficacy in apathy management thus far, there is increasing interest in employing nonpharmacological interventions to reduce apathy [16], including solutions based on new ICTs, such as virtual reality and serious games [14,15]. In this RCT, we tested the efficacy of MeMo, a Web-based app for memory, attention, and mental flexibility training (MeMo group) by comparing it with treatment as usual (control group) in terms of cognitive function and behavioral symptoms, particularly apathy. The results collected from participants with mild and major NCDs showed small but significant differences in attentional and executive function tests and apathy over a 3-month training period. However, these positive effects were only observed for participants who regularly used the app, as indicated in the study protocol (at least 2 days per week; MeMo active subgroup). Specifically, participants in the nonactive MeMo group showed a decline in performance regarding attention and a slight increase in apathy over the 3-month training period, whereas participants in the active MeMo group showed stable results over time. This kind of “dose effect” of cognitive training has been previously observed. In a recent review, Sood et al [32] reported two studies in older subjects suggesting a relationship between the efficacy of the intervention and the play time [33] or the number of gaming sessions over a period of 2 months [34]. Similarly, improvements in attention and executive function have been previously reported following cognitive training. For instance, Sood et al [32] in their recent literature review found that out of 18 studies, eight studies reported a great improvement in attention/working memory. In addition, nine of the reviewed studies assessed depression, a behavioral symptom, as an outcome variable. Four of these studies reported a great reduction in depression among individuals with mild cognitive disorders. Ge et al [7] also reported this noncognitive outcome for depression and anxiety. In this study, we assessed apathy. Apathy is a disorder of motivation defined as a quantitative reduction in goal-directed activity in comparison with the patient’s previous level of functioning [11,35]. Symptoms must persist for at least 4 weeks and affect at least two of the three apathy dimensions (behavior/cognition, emotion, and social interaction). Apathy and depression show some overlap in terms of prevalence and brain circuits, but they can be differentiated [36]. Thus, apathy should be measured as an independent outcome variable. In this study, after 12 weeks, the active MeMo participants were significantly less apathetic as compared with the nonactive MeMo participants (*P*=.02). This is in line with the findings of preliminary studies performed by our group [15,37] and suggests the interest of employing cognitive training delivered through ICT to increase participants’ motivation to train [22]. As indicated by Booth et al [38], motivation is a core element of the program theory underlying fall prevention interventions in older adults with cognitive impairment. Within the motivation component of this program, two key mechanisms (perceived benefit and support) were shown to influence the extent to which an older adult with cognitive impairment is motivated to undertake an exercise-based intervention. MeMo was designed to increase the intrinsic motivation to keep exercising thanks to several features [22]. First, the game interface was specifically designed for the target population of older adults with cognitive impairment (eg, simplified graphical user interface, simplified instructions with regular reminders, and clear game rules) and was thus easily usable for participants in this study. In addition, the level of difficulty of the exercises was dynamically adapted to the participant’s performance in order to provide an errorless type of training [39] and to keep the participant in a “challenge zone” [20]. Finally, MeMo was tested on a tablet instead of a computer to reduce constraints associated with the use of nonfamiliar technological interfaces, such as a mouse and keyboard, which are often perceived as difficult to use by older adults [20].

Although the exercise design was meant to increase motivation, it should be noted that only 41% (9/22) of the participants used the app as indicated in the guidelines of the research protocol, and the others exercised less. A better training adherence could have been accomplished by scheduling regular in-person phone meetings or discussions with clinicians. This would have probably increased the number of participants that adhered to the protocol guidelines. Indeed, it has been previously suggested that the presence of a clinician is a key factor in determining ICT-based treatment adherence [37]. However, in the RCT, we did not want to add external motivation factors to be able to assess the app efficacy itself. Future studies should test if adding regular meetings with clinicians or support by a caregiver can improve treatment adherence.

Our results are promising; however, the study has several limitations. The major limitation of this study is the small number of participants. Initially, we aimed to include 40 patients in each group considering participant drop out and possible nonexploitability of data. In reality, we managed to include only 46 participants in the time period of the study, although the research was proposed to almost 100 participants who met the inclusion criteria. The main reason for refusal to participate in the trial was concern over the use of new technologies. The mean age of the included participants was 79.4 years. It is reasonable to estimate that in a country like France, the proportion of refusal would have been lower among younger subjects, but this situation will rapidly change. Indeed, people aged over 50 years represent a non-negligible percentage of digital gamers (11% in the United States) according to the 2018 Entertainment Software Association report [40], and this percentage is increasing every year. Playing games is recognized as a source of human enjoyment. Gamified training has been shown to increase motivation, positive mood, and compliance, and therefore, it could substantially drive cognitive benefits [41]. However, the quality of evidence is currently low [6], and the factors boosting motivation to play among older adults are still not completely known. To maximize future brain-gaming tools, it is critical to be able to appreciate what aspects are promising and what aspects are not promising for possible cognitive improvements among older adults with cognitive impairments.

A second limitation of this study is that differences in cognitive performance between active and nonactive MeMo participants were quite small and were limited to the domains of attention and executive functions. Although this is consistent with previous studies [32], it questions the generalizability of the effects of cognitive training to real-life cognitive functions and the general impact on activities of daily living. It should be noted that technology-based games and apps are usually employed in the context of multidomain interventions, and this may increase their benefits in real-life settings [2,3].

In summary, the main result of this study is that the cognitive and behavioral efficacies of a Web-based cognitive training app can be observed only after regular use. In a clinical setting, this points out the importance of reminding about regular use at the moment of prescription and possibly the requirement of regular follow-ups to check prescription adherence.

## Appendix

Multimedia Appendix 1 CONSORT-eHEALTH Checklist (V 1.6.1).

## Abbreviations

AI: Apathy Inventory
DSST: Digit Symbol Substitution Test
FAB: Frontal Assessment Battery
ICT: information and communication technology
IQCODE: Informant Questionnaire on Cognitive Decline in the Elderly
MeMo: Memory Motivation
MMSE: Mini-Mental State Examination
NCD: neurocognitive disorder
NPI: Neuropsychiatric Inventory
RCT: randomized controlled trial
TMT A: Trial Making Test A
